# Photosynthetic Performance Across Urban Green Spaces Within a University Campus Ecosystem

**DOI:** 10.3390/plants15162491

**Published:** 2026-08-17

**Authors:** Bar Cristina, Cosmin Alin Popescu, Adina Horablaga, Giancarla Velicevici, Dorin Camen

**Affiliations:** 1 Doctoral School Engineering of Plant and Animal Resources, University of Life Sciences King Michael I, 119 Calea Aradului, 300645 Timișoara, Romania; cristina.bar2004@gmail.com; 2 Department of Sustainable Development and Environmental Engineering, Faculty of Agriculture, University of Life Sciences King Mihai I from Timisoara, 300645 Timisoara, Romania; cosmin_popescu@usvt.ro (C.A.P.); adinahorablaga@usvt.ro (A.H.); 3 Department of Genetic Engineering, Faculty of Engineering and Applied Technologies, University of Life Sciences King Mihai I from Timisoara, 300645 Timisoara, Romania; giancarlavelicevici@gmail.com

**Keywords:** urban green spaces, photosynthetic performance, urban ecology, vegetation structure, urban horticulture, ecological assessment, university campus, ecosystem functionality, climate adaptation, sustainable urban planning

## Abstract

Urban green spaces play a fundamental role in maintaining ecological stability, supporting biodiversity, regulating urban microclimates, and improving human wellbeing. Because photosynthetic activity is a key indicator of vegetation functionality and environmental adaptation, this study evaluated the photosynthetic performance of vegetation across eight urban green spaces within the campus of the University of Life Sciences “King Michael I” in Timișoara, Romania. Vegetation structure, spatial organization, and estimated photosynthetic rates of selected ornamental species were comparatively analyzed under the temperate climatic conditions of the study area. Two-factor analysis of variance (ANOVA) revealed that the spatial characteristics of the investigated green spaces had a highly significant effect on photosynthetic variability (F(7, 42) = 6.783; *p* = 0.000021), explaining approximately 46% of the total variance, whereas species identity accounted for only 13.3% and showed no statistically significant influence (*p* = 0.052863). The highest photosynthetic performance was recorded in structurally diverse green spaces characterized by dense tree canopy and balanced woody–herbaceous vegetation, whereas the lowest values occurred in highly urbanized areas with limited vegetation cover. These findings demonstrate that the structural organization and vegetation composition of urban green spaces strongly influence photosynthetic performance and support the use of integrated ecological assessment as a decision-support tool for sustainable and climate-resilient urban green infrastructure planning.

## 1. Introduction

Urban green spaces represent essential structural and functional components of contemporary cities, contributing significantly to ecological stability, climate regulation, biodiversity conservation and human well-being [1,2,3,4,5,6]. In highly anthropized environments, vegetation performs critical ecosystem services through carbon sequestration, oxygen production, temperature regulation, particulate matter retention and the improvement of microclimatic conditions. The ecological efficiency of urban vegetation is closely linked to photosynthetic performance, which represents one of the most important physiological indicators of plant functionality and adaptation capacity within urban ecosystems [1,3].

The increasing intensity of urbanization has generated substantial pressure on urban ecological systems, leading to the fragmentation of vegetated areas, reduction in biodiversity, alteration of local climatic conditions and degradation of ecosystem functionality [3,5,7,8,9]. In this context, urban green spaces are no longer evaluated exclusively through quantitative indicators such as total area or green surface per inhabitant but increasingly through qualitative and functional criteria associated with vegetation structure, accessibility, ecological value and environmental performance [3,10,11,12,13,14].

Urban green spaces are increasingly evaluated in relation to their ecological quality and functional performance. Contemporary urban ecology emphasizes that the environmental value of green infrastructure depends strongly on vegetation structure, species diversity, spatial connectivity and ecosystem resilience. Consequently, urban landscapes characterized by heterogeneous vegetation patterns and balanced structural composition may provide substantially higher ecological benefits than simplified ornamental green areas with limited biological complexity. The ecological quality of urban green spaces therefore represents a critical component in climate adaptation strategies, biodiversity conservation and the long-term sustainability of urban ecosystems [3,7,10,13,15].

The qualitative assessment of urban green spaces has become an important research direction within urban ecology, landscape architecture and horticultural sciences. Previous studies demonstrated that vegetation composition, spatial configuration and structural diversity strongly influence the ecological functionality of urban landscapes. In particular, the composition of the vegetal framework, the proportion between woody and herbaceous species, and the complexity of vegetation layers can affect ecosystem stability and physiological plant responses.

Urban vegetation is continuously exposed to environmental stress factors including elevated temperatures, soil compaction, altered hydrological regimes, atmospheric pollution and anthropogenic disturbance [16,17,18]. Under such conditions, photosynthesis becomes a sensitive physiological indicator reflecting the adaptive response of plant species to urban environmental quality. Consequently, the analysis of photosynthetic performance provides valuable information regarding the ecological functionality and sustainability potential of urban green infrastructures.

Photosynthesis additionally represents one of the fundamental ecological processes supporting ecosystem stability within urban environments. Through carbon assimilation, oxygen production and energy transfer within trophic systems, photosynthetic activity contributes directly to climate regulation and environmental quality. In urban ecosystems, the photosynthetic capacity of vegetation is closely associated with ecosystem services such as urban cooling, atmospheric pollutant mitigation and carbon sequestration. Consequently, the evaluation of photosynthetic performance may provide valuable insights into the functional status, adaptive capacity and ecological efficiency of urban green infrastructures under increasing anthropogenic pressure [1,3,4,19,20].

Beyond its physiological significance, the assessment of photosynthetic performance represents a valuable decision-support tool for urban planning and green infrastructure management [21]. Spatial evaluation of photosynthetic activity enables the identification of vegetation sectors with high ecological functionality as well as areas affected by environmental stress, thereby supporting evidence-based interventions aimed at improving ecosystem service delivery. Furthermore, integrating photosynthetic indicators into landscape assessment may assist urban managers in optimizing vegetation composition, prioritizing restoration measures, enhancing climate resilience, and increasing the long-term sustainability of urban green infrastructure under rapidly changing environmental conditions [10,21,22,23,24,25].

Several methodological approaches have been developed to evaluate photosynthetic performance in plant communities. Direct physiological measurements, such as gas-exchange analysis using portable photosynthesis systems and chlorophyll fluorescence techniques, are widely employed to quantify carbon assimilation and photosynthetic efficiency under field conditions [26]. Complementary approaches include remote sensing, spectral vegetation indices, and eco-physiological modeling, which enable the assessment of vegetation performance across larger spatial scales. The selection of an appropriate methodology depends on the objectives of the study, the spatial extent of the investigated ecosystem, and the availability of experimental data. In studies focused on comparative ecological assessment, estimated photosynthetic performance based on species-specific physiological characteristics reported in the literature may provide a consistent framework for evaluating functional differences among urban green spaces under standardized environmental conditions.

Most existing studies concerning urban green spaces focus either on ecosystem services or on floristic and landscape composition, while relatively limited research has investigated the relationship between the qualitative structure of urban green spaces and photosynthetic performance at the ecosystem scale [3,5,10,11,12,13,14]. Furthermore, comparative analyses between multiple functional green areas within the same urban ecosystem remain insufficiently explored, especially in Eastern European university campus environments. University campuses constitute highly relevant experimental models for urban ecological research because they integrate heterogeneous green spaces with different landscape structures, vegetation compositions, management practices and anthropogenic pressures within a relatively controlled territorial framework. These characteristics allow comparative assessment of vegetation performance under differentiated spatial and ecological conditions [13,15,27].

The present study investigates the photosynthetic performance of vegetation distributed across eight urban green spaces located within the campus of the University of Life Sciences “King Michael I” from Timișoara. The research explores the relationship between green space structure and photosynthetic activity by integrating vegetation composition, spatial distribution patterns and average annual photosynthetic rates estimated under temperate climatic conditions specific to Timișoara.

The main hypothesis of the study is that the qualitative characteristics of urban green spaces exert a stronger influence on photosynthetic performance than species identity alone [3,7,10]. In this context, the research aims to investigate how vegetation structure, spatial organization and green space composition influence the ecological functionality of urban plant communities.

The objectives of the study were as follows:

1. To evaluate the photosynthetic performance of selected plant species across eight urban green spaces;
2. To compare photosynthetic variability between distinct functional zones within the university campus ecosystem;
3. To determine the relative contribution of spatial and species-related factors to photosynthetic variability;
4. To provide a functional ecological perspective regarding the quality assessment of urban green spaces.

This study contributes to the growing body of research on urban ecological functionality by integrating vegetation structure analysis with indicators of photosynthetic performance to assess the ecological quality of urban green spaces. The results demonstrate that the structural organization and composition of urban vegetation exert a greater influence on photosynthetic performance than species identity alone, highlighting the importance of vegetation diversity and spatial heterogeneity in enhancing ecosystem functionality. These findings provide a scientific basis for supporting sustainable urban planning, biodiversity conservation, and climate-resilient green infrastructure management.

## 2. Materials and Methods

### 2.1. Study Area

The study was conducted within the campus of the University of Life Sciences “King Michael I” from Timișoara, Romania. The university campus represents a heterogeneous urban ecosystem characterized by multiple categories of landscaped green spaces differing in vegetation structure, floristic composition, spatial organization and functional use.

**Figure 1:**
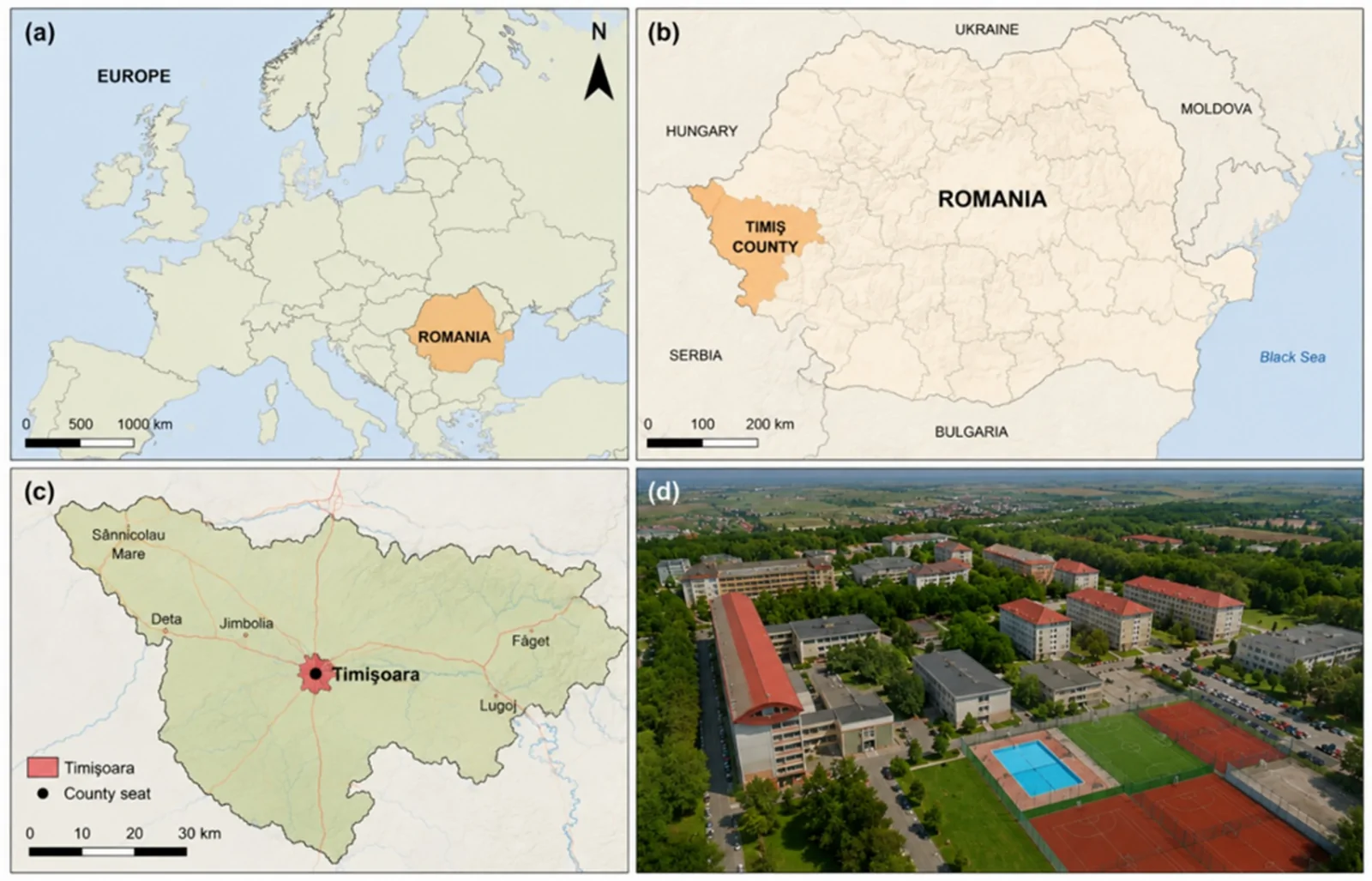
Geographical location of the study area: (**a**) position of Romania within Europe; (**b**) location of Timiș County within Romania; (**c**) location of Timișoara within Timiș County; (**d**) aerial view of the University of Life Sciences “King Michael I” campus, where the study was conducted.

The campus of the University of Life Sciences “King Michael I” from Timișoara is located in the eastern part of Timișoara, Romania, within a dynamically developing urban sector representative of the recent expansion of cities in Western Romania. The study area is situated in a transitional zone between the consolidated urban core and newer residential developments, characterized by variable building densities and interspersed green spaces. Owing to its spatial configuration and extensive vegetation cover, the university campus constitutes an important green infrastructure component within the urban landscape of Timișoara. The geographical location of the study area is presented in Figure 1.

**Figure 2:**
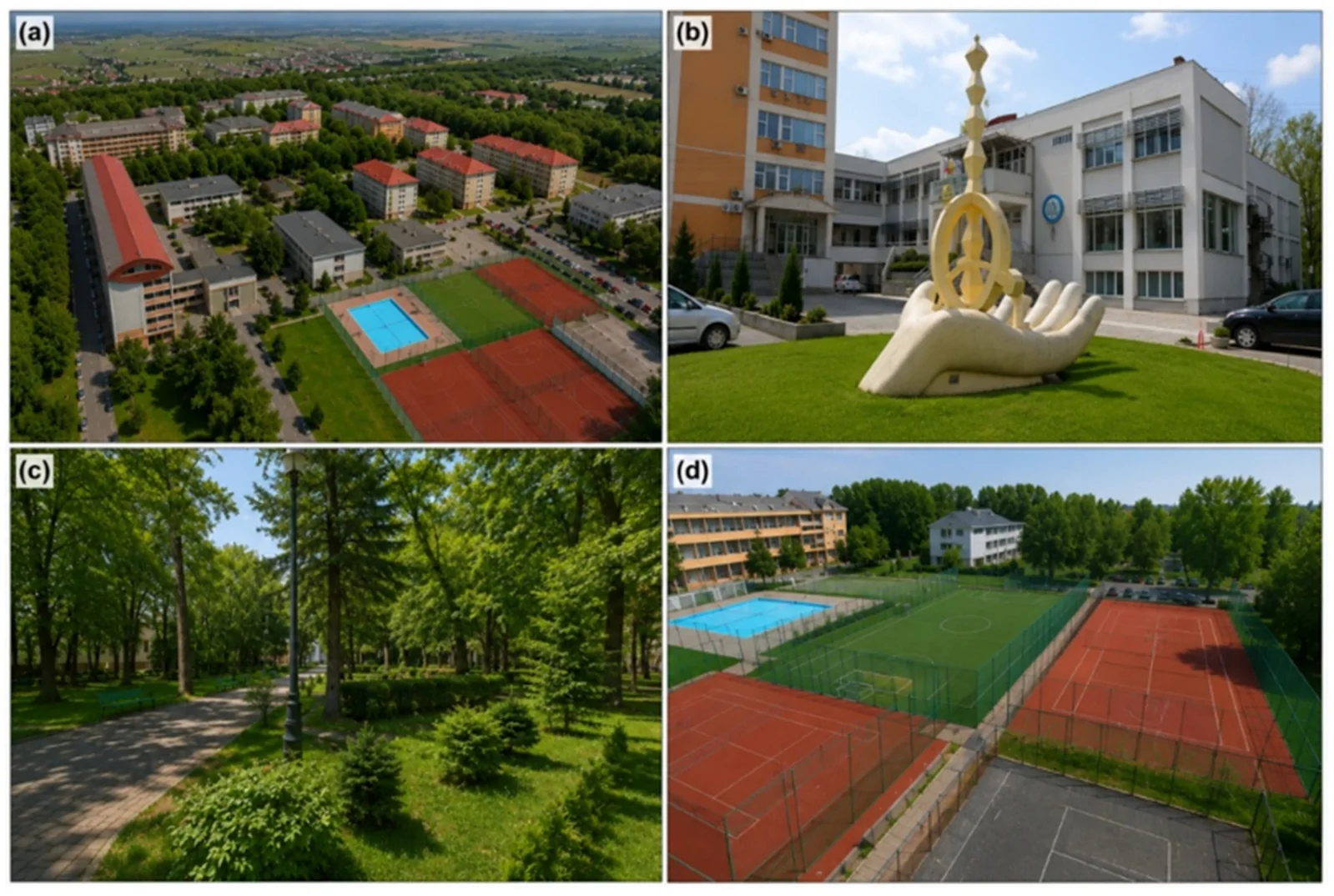
Representative views of the University of Life Sciences “King Michael I” campus illustrating the main academic buildings, urban green infrastructure, and recreational facilities surrounding the investigated vegetation. Panels show: (**a**) aerial view of the university campus; (**b**) main academic building; (**c**) representative urban green space; and (**d**) sports and recreational facilities integrated within the campus landscape.

From a geographical perspective, the study area is located within the Western Plain, a low-altitude relief unit favorable both for rapid urbanization and for the development of herbaceous and woody vegetation. The predominantly flat topography enabled the organization of the campus as an open spatial ensemble, characterized by wide pedestrian alleys, continuous green areas and mature trees forming internal ecological corridors. An important component of the urban context is represented by the diversity of green space typologies existing within the campus. The area includes carefully designed landscapes with regularly maintained lawns and ornamental trees, as well as less intensively managed surfaces where spontaneous vegetation has naturally established. This coexistence provides an appropriate framework for the functional comparison between planted and spontaneous vegetation, as well as for evaluating the extent to which urban management practices influence ecological performance. Representative views of the university campus, illustrating its urban green infrastructure and the environmental context of the investigated vegetation, are presented in Figure 2.

Eight representative green spaces distributed throughout the campus were selected for analysis. The investigated areas included thematic and functional landscape sectors characterized by distinct vegetal compositions and different proportions of woody and herbaceous vegetation. The analysed zones were identified as follows: Zone 1, Zone 2, Zone 4, Zone 5, Zone 7, Zone 8, Zone 9 and Zone 10. The total vegetated surface included in the study was 12,261 m^2^. Each zone presented different structural characteristics regarding vegetation density, species composition, ornamental typology and estimated spatial occupation of vegetal elements.

### 2.2. Climatic Conditions

Timișoara is located in the western part of Romania and is characterized by a temperate–continental climate with moderate sub-Mediterranean influences. The local climate is generally defined by relatively mild winters, warm summers and moderate annual precipitation.

The photosynthetic assessment was performed under temperate climatic assumptions using the average annual temperature specific to the Municipality of Timișoara as the main climatic reference parameter. The use of a common climatic baseline allowed the comparative evaluation of photosynthetic behavior among species and across urban green spaces under standardized environmental conditions.

### 2.3. Vegetation Structure and Plant Material

The vegetation analysis included woody and herbaceous species distributed across the eight selected green spaces. Species inventory and quantitative distribution were extracted from the vegetation master database developed for the analyzed campus sectors.

For each study zone, the seven most abundant plant species were selected for comparative analysis, irrespective of their classification as woody or herbaceous vegetation.

In Zone 1, the selected species were represented exclusively by woody ornamental vegetation, including *Euonymus* globular forms, *Ilex* globular forms, *Nandina domestica*, *Berberis thunbergii* ‘Red Rocket’, *Berberis thunbergii* ‘Golden Rocket’, shrub-form *Euonymus*, and *Nandina domestica* ‘Fire Power’.

In Zone 2, both herbaceous and woody species were included, namely *Yucca* sp., *Rhapidophyllum hystrix*, *Trachycarpus fortunei*, *Dypsis lutescens*, *Phyllostachys nigra*, *Musa basjoo*, *Sempervivum* spp., *Soleirolia soleirolii*, *Prunus laurocerasus*, and *Photinia × fraseri* ‘Red Robin’.

Zone 4 was characterized predominantly by herbaceous ornamental vegetation, represented by *Lavandula* spp., *Echinacea purpurea* ‘Magnus’, *Salvia splendens*, *Bellis perennis*, *Myosotis alpestris*, *Viola × wittrockiana*, and perennial *Salvia officinalis*.

In Zone 5, the selected vegetation included *Dicentra spectabilis*, *Asplenium trichomanes*, *Athyrium filix-femina*, hydrangeas (*Hydrangea* spp.), *Heuchera* spp., *Hosta* spp., and *Rhododendron* spp.

Zone 7 included mainly shade-adapted herbaceous vegetation represented by *Paeonia* spp., *Phyllitis scolopendrium*, *Pteridium aquilinum*, *Athyrium niponicum* ‘Metallicum’, *Matteuccia struthiopteris*, and *Asplenium trichomanes*, together with *Juniperus* spp. as the representative woody species.

In Zone 8, the vegetation structure included both woody and herbaceous ornamental species, namely *Rosa* spp., *Hosta* spp., *Aster* spp., *Rudbeckia* spp., *Echinacea* spp., *Monarda* spp., and *Lupinus* spp.

Zone 9 combined woody ornamental vegetation such as *Lavandula* spp., *Ligustrum* spp., and dwarf *Rosa* cultivars with herbaceous species represented by *Aster* spp., *Rudbeckia* spp., *Echinacea* spp., and *Monarda* spp.

In Zone 10, the selected species included *Thuja occidentalis* ‘Columnaris’, *Thuja occidentalis* ‘Danica’, *Rosa Polyantha* ‘Schwarze Madonna’, *Rosa Polyantha* ‘Grand Gala’, *Rosa Polyantha* ‘Iceberg’, *Ocimum basilicum*, and *Thymus* spp.

For each zone, the following parameters were considered: total number of plant specimens; estimated occupied surface; spatial distribution of species; photosynthetic rate for each species.

The vegetal framework included a diverse range of ornamental trees, shrubs, perennial herbaceous plants, grasses, ferns and decorative species frequently used in urban landscape architecture. Species selection reflected the functional and aesthetic specificity of each landscape sector.

The estimated surface occupied by each species was determined proportionally according to plant abundance and spatial distribution within each analyzed zone. For species characterized by distinct morphological traits and different spatial development patterns, specific estimation coefficients were applied in order to obtain a more realistic ecological representation of vegetation coverage.

### 2.4. Estimation of Photosynthetic Performance

The photosynthetic performance was estimated for the selected plant species under temperate climatic conditions using the average annual temperature specific to the Municipality of Timișoara as the main climatic reference parameter. The estimation process considered the ecological and physiological characteristics of the analyzed species together with their adaptation to urban environmental conditions. The estimated photosynthetic values were comparatively derived based on species-specific physiological characteristics reported in the specialized horticultural and eco-physiological literature and correlated with plant ecological adaptability under temperate climatic conditions. The methodological approach was not intended to provide direct gas-exchange measurements but rather to establish a comparative ecological framework for evaluating relative photosynthetic functionality across urban green spaces under standardized environmental assumptions.

Photosynthetic performance was analyzed comparatively at the levels of individual species, urban green spaces, and the overall campus ecosystem.

The comparative approach allowed the identification of spatial variability patterns in photosynthetic performance across the university campus ecosystem.

The analysis also considered the relationship between vegetation structure and ecological functionality, particularly regarding the influence exerted by species composition and green space configuration on photosynthetic dynamics.

### 2.5. Statistical Analysis

The statistical analysis aimed to evaluate the influence of spatial distribution and species-related variability on photosynthetic performance. Statistical analyses were performed using IBM SPSS Statistics version 27.0. A two-factor analysis of variance (ANOVA) was performed in order to assess the effects of the spatial factor (urban green space) and the species factor (plant variants) on the variability in photosynthetic rate values.

The significance of differences between means was evaluated using Duncan’s Multiple Range Test and Student’s *t*-test. In addition, hierarchical clustering methods were used to identify similarities and spatial associations between the analyzed zones according to photosynthetic performance characteristics.

The statistical interpretation focused on determining the contribution of vegetation structure and spatial organization to photosynthetic variability within the urban ecosystem framework.

## 3. Results

### 3.1. General Variability in Photosynthetic Performance Across Urban Green Spaces

**Figure 3:**
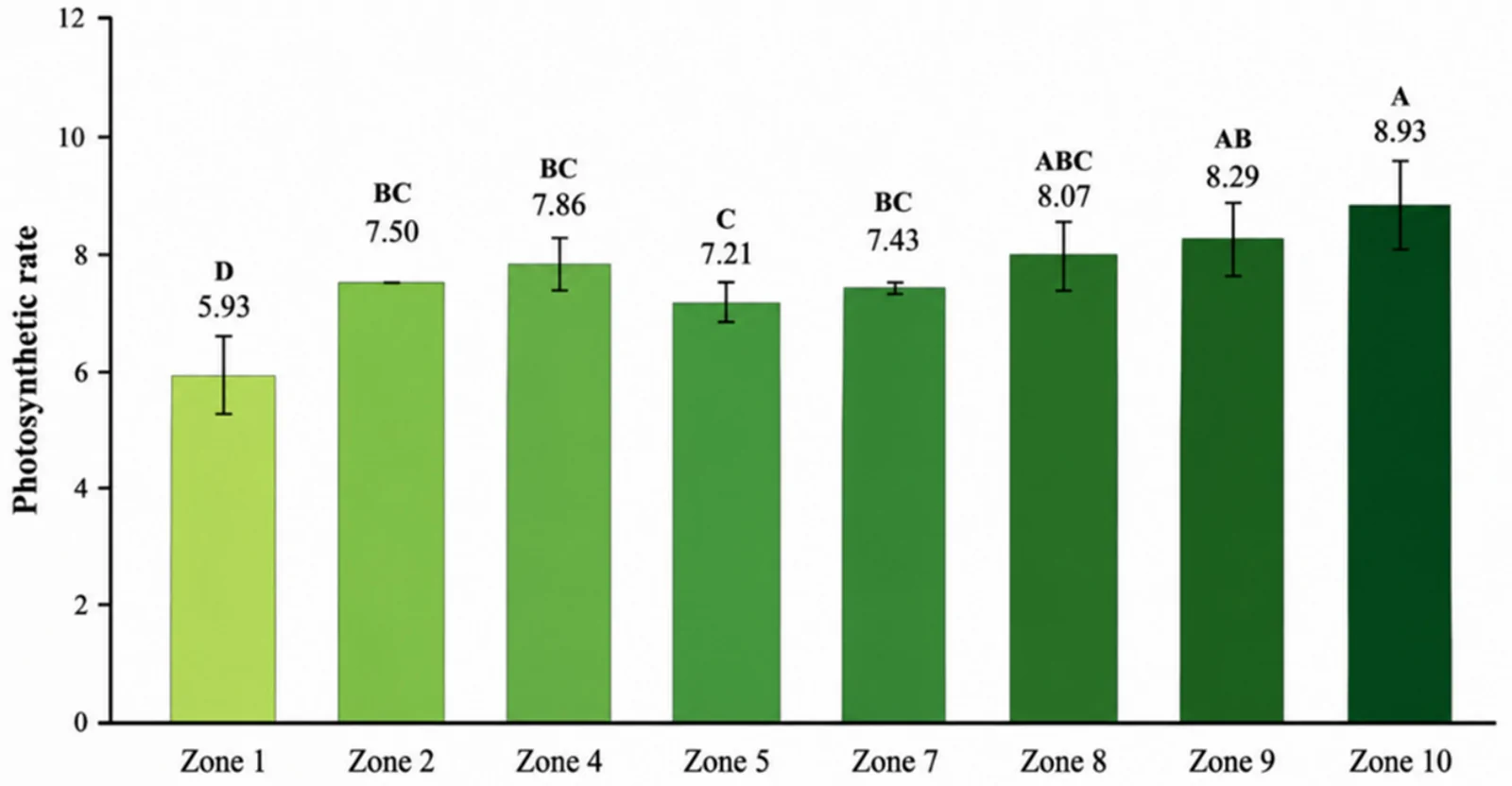
Mean photosynthetic rates (±SE) recorded in the eight analyzed urban green spaces. Different letters indicate statistically significant differences according to Duncan’s multiple range test (*p* < 0.05).

The comparative analysis of the eight investigated urban green spaces revealed substantial variability in photosynthetic performance across the university campus ecosystem. The results indicate that differences in the spatial organization and structural characteristics of the analyzed green spaces were associated with significant variation in estimated photosynthetic rates. Photosynthetic performance varied considerably among the investigated zones, reflecting differences in vegetation composition, structural organization, and ecological complexity. The highest estimated photosynthetic rate was recorded in Zone 10 (8.93), whereas the lowest value was observed in Zone 1 (5.93). The remaining zones exhibited intermediate values, reflecting gradual differences in vegetation structure and ecosystem characteristics. Urban green spaces characterized by greater structural heterogeneity and more diverse vegetation assemblages generally exhibited higher estimated photosynthetic performance than areas dominated by simplified ornamental vegetation. Table 1 and Figure 3 summarize the comparative distribution of photosynthetic rates across the analyzed urban green spaces.

Values are presented as mean ± standard error (SE) together with the corresponding 95% confidence intervals. Standard error and confidence intervals were not estimated for Zone 2 because no within-group variability was observed in the dataset. Differences among study zones were subsequently evaluated using one-way ANOVA followed by Duncan’s multiple range test.

These results suggest that urban green spaces characterized by greater compositional heterogeneity and more diverse vegetation architecture provide more favorable conditions for maintaining higher levels of physiological activity.

The marked contrast between Zones 1 and 10 can be primarily attributed to differences in vegetation composition, structural complexity, and spatial extent. Zone 1 consisted exclusively of woody ornamental species and exhibited a relatively homogeneous vegetation structure, with woody vegetation accounting for 80.57% of the analyzed area. In contrast, Zone 10 comprised both woody and herbaceous vegetation, including columnar and dwarf conifers, rose cultivars, and aromatic herbaceous species, resulting in greater vegetation diversity and a more balanced woody–herbaceous distribution (68.47% and 31.53%, respectively). Zone 10 also represented the largest investigated green space (approximately 4413.3 m^2^), whereas Zone 1 covered 1358.5 m^2^. These structural characteristics likely enhanced spatial heterogeneity, vegetation stratification, and the diversity of physiological responses within the vegetation community, thereby contributing to the higher estimated photosynthetic performance observed in Zone 10. Because microclimatic and edaphic variables were not directly measured, the potential influence of light availability, soil moisture, temperature, irrigation, and anthropogenic disturbance should be regarded as plausible explanatory factors rather than experimentally demonstrated causes.

### 3.2. Influence of Spatial Factor on Photosynthetic Rate

The statistical analysis performed for the spatial factor represented by the eight analyzed urban green spaces revealed significant variability in photosynthetic performance among the investigated zones. The recorded photosynthetic values ranged from 5.93 in Zone 1 to 8.93 in Zone 10, indicating substantial ecological differences between the analyzed green spaces.

The lowest photosynthetic value was identified in Zone 1, while the highest value characterized Zone 10. Intermediate values were recorded for the remaining zones, namely 7.50 in Zone 2, 7.86 in Zone 4, 7.21 in Zone 5, 7.43 in Zone 7, 8.07 in Zone 8, and 8.29 in Zone 9. These results suggest that vegetation structure, spatial organization, and plant diversity substantially influenced ecosystem-level photosynthetic performance. The analysis of variance revealed that the differences between the investigated zones were statistically significant according to the F-test (*p* = 0.00002). Overall, the statistical analysis confirmed the strong influence exerted by the spatial factor on photosynthetic performance.

Student’s *t*-test further identified statistically significant differences between several study zones and the overall experimental mean. Zone 10 exceeded the experimental mean by 1.3 units and was statistically significant at the 1% probability level (**), while Zone 1 recorded a significantly lower value compared to the experimental mean, showing a negative difference of −1.72 units and a highly significant deviation (*). The remaining zones showed non-significant differences relative to the experimental average.

**Figure 4:**
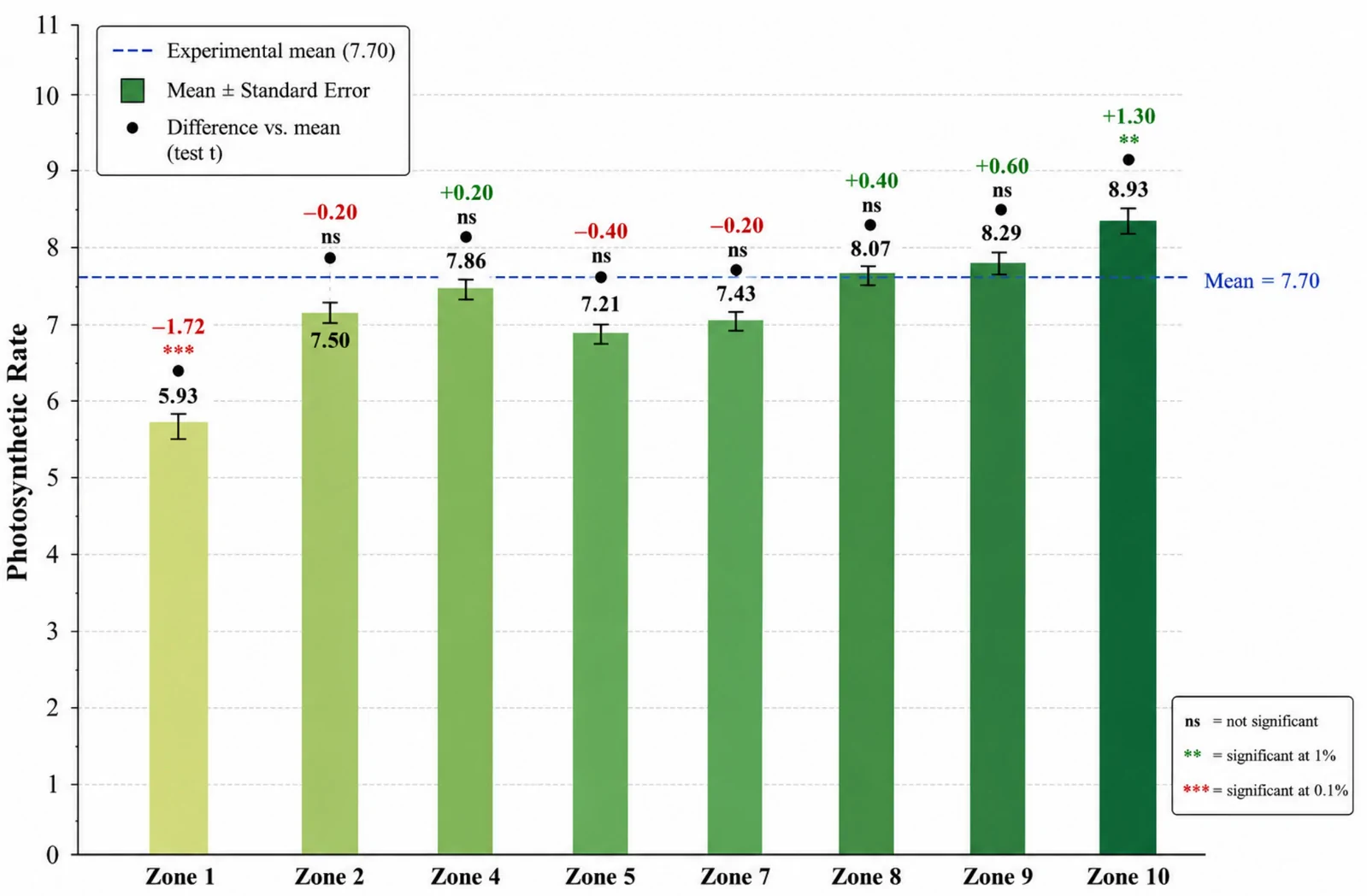
Student’s *t*-test analysis of photosynthetic rate across the analyzed urban green spaces.

Table 2 and Figure 4 summarize the statistical influence of the spatial factor on photosynthetic performance across the analyzed urban green spaces.

**Figure 5:**
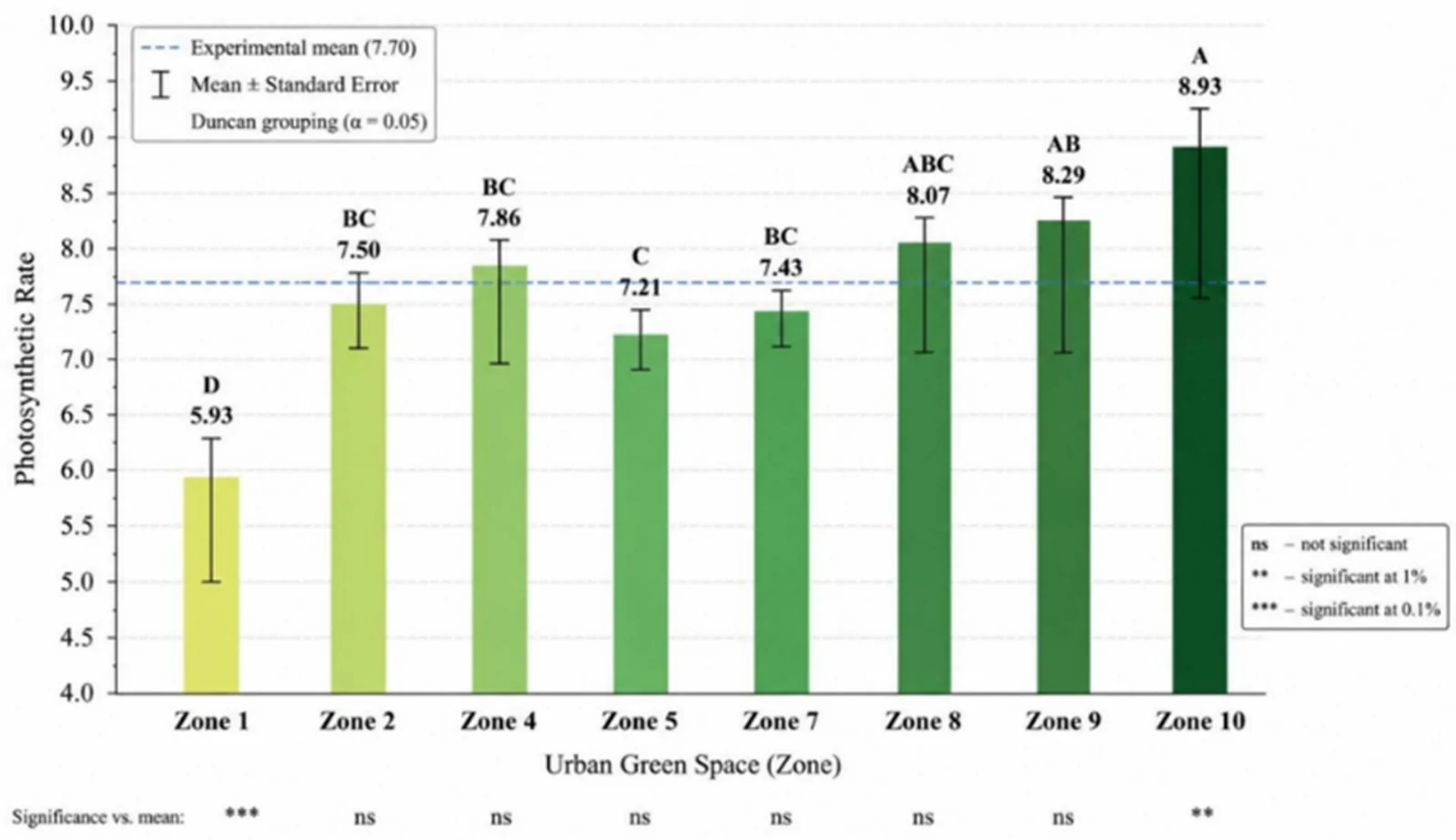
Duncan grouping of photosynthetic rate across the analyzed urban green spaces. Different letters indicate statistically significant differences at *p* < 0.05.

The Duncan multiple range test classified the analyzed zones into four statistical classes (A–D). Zone 10 was included in class A and displayed the highest estimated photosynthetic rate, significantly differing from most other zones, except Zones 8 and 9, which also contained the letter A within their statistical grouping. In contrast, Zone 1 formed a distinct class (D), characterized by the lowest photosynthetic performance and statistically differentiated from all other investigated zones. Zones 2, 4 and 7 were grouped within the same statistical class (BC), indicating relatively similar photosynthetic responses among these green spaces. Table 3 and Figure 5 summarize the statistical influence of the spatial factor on photosynthetic performance across the analyzed urban green spaces.

### 3.3. Influence of Plant Species on Photosynthetic Variability

The analysis of the species factor revealed notable variability in photosynthetic performance among the investigated vegetation variants. The estimated photosynthetic values ranged from 7.06 for variant V6 to 8.31 for variant V1, indicating differentiated physiological responses among the analyzed plant assemblages.

The highest photosynthetic value was recorded for variant V1 (8.31), followed by variant V3 (8.13), while the lowest value characterized variant V6 (7.06). Intermediate values were identified for the remaining variants, namely 7.19 for V2, 7.88 for V4, 7.69 for V5, and 7.31 for V7. These differences suggest that species composition and vegetation structure considerably influenced the overall photosynthetic performance of the analyzed urban green spaces.

The Duncan multiple range test grouped the analyzed variants into three statistical homogeneity classes (A–C). Variant V1 formed the highest statistical class (A), while variant V6 belonged to class C, corresponding to the lowest photosynthetic performance. Variants V3, V4, and V5 were positioned within intermediate statistical classes (AB and ABC), indicating relatively similar physiological responses among these plant variants.

The observed patterns further support the importance of species composition in determining ecosystem-level photosynthetic variability and reinforce the influence of vegetation structure on the ecological performance of urban green spaces.

**Figure 6:**
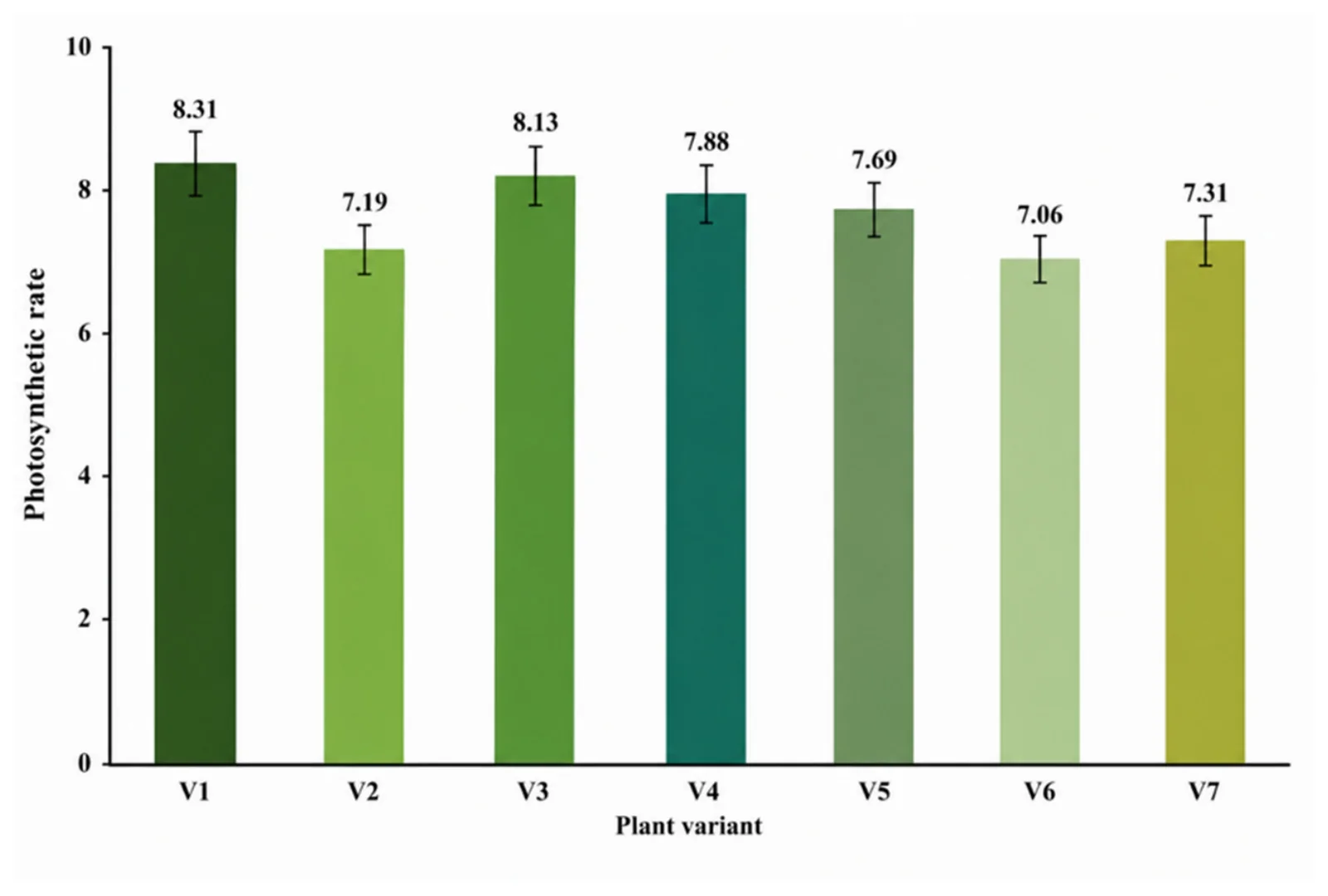
Mean photosynthetic rates (±SE) recorded for the seven analyzed plant variants.

**Figure 7:**
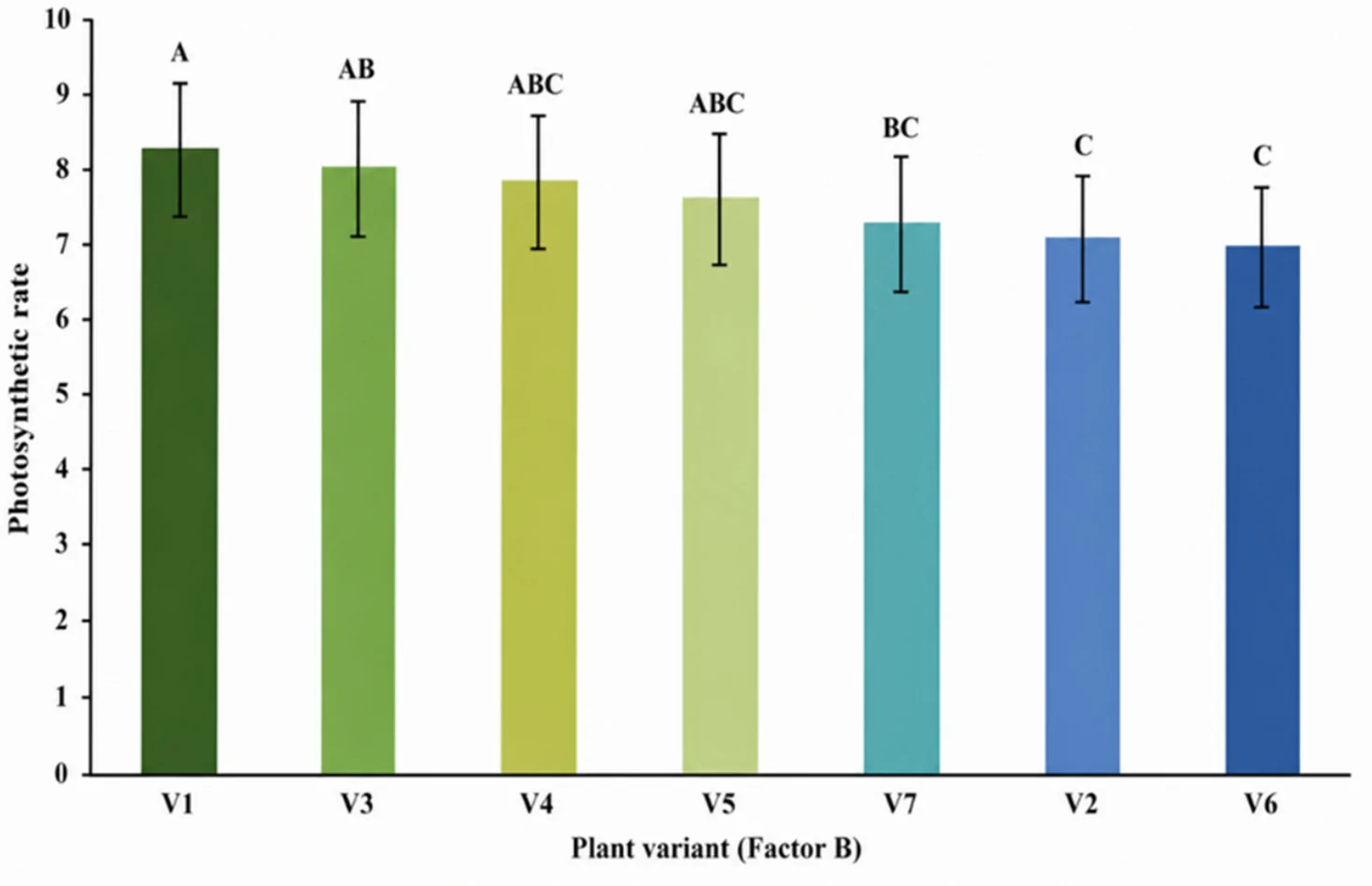
Duncan multiple range test showing the statistical grouping of mean photosynthetic rates (±SE) among the analyzed plant variants. Different letters indicate statistically significant differences at *p* < 0.05.

Table 4 and Figure 6 summarize the comparative photosynthetic values recorded for the analyzed plant species, while Table 5 and Figure 7 present the statistical grouping obtained through the Duncan multiple range test.

### 3.4. Cluster Analysis of Spatial and Species Factors

Cluster analysis was performed for both experimental factors in order to evaluate the similarity relationships between the analyzed urban green spaces and plant variants according to their photosynthetic performance. The dendrograms were generated using the single linkage clustering method based on Euclidean distances.

**Figure 8:**
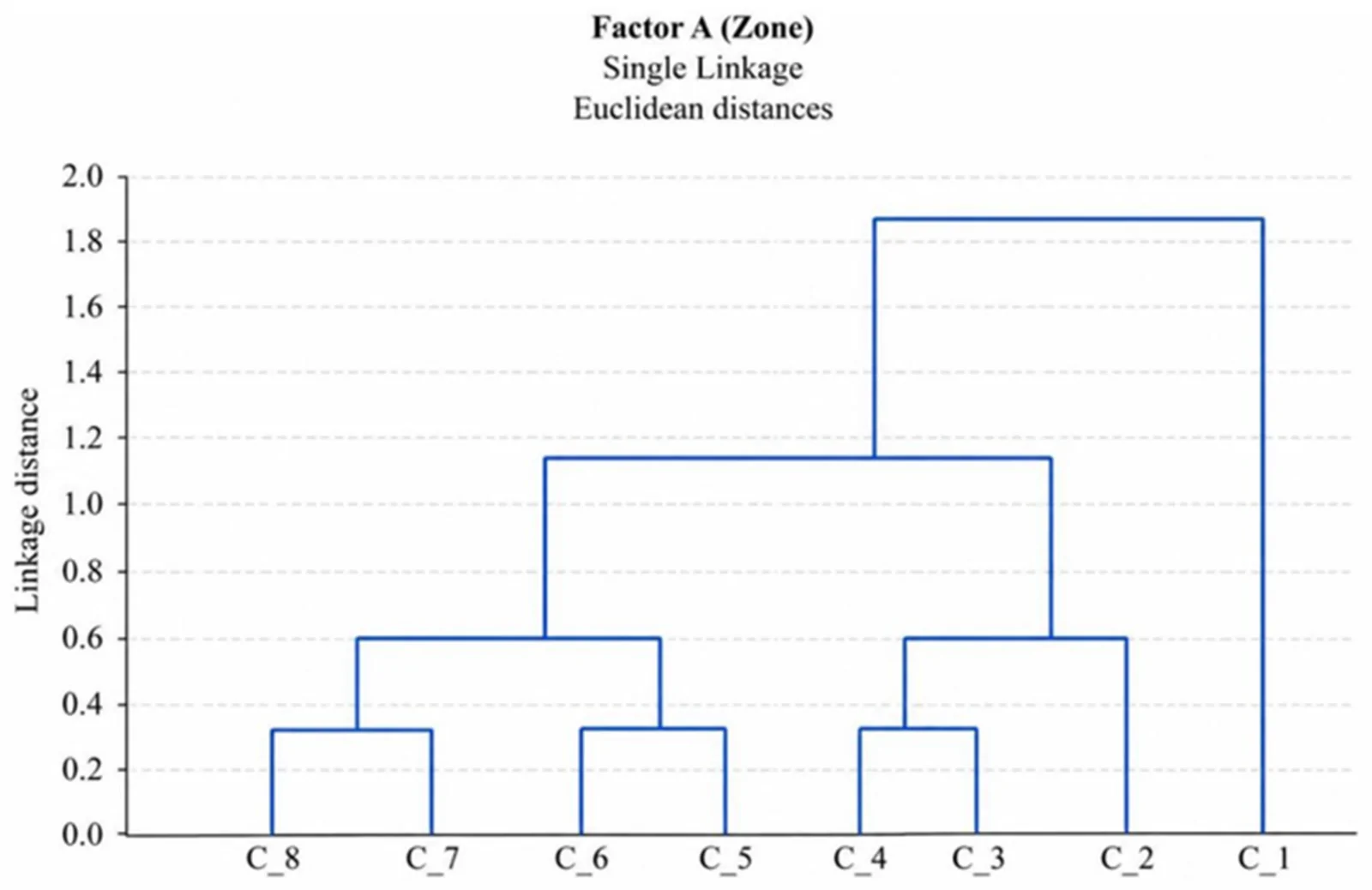
Cluster dendrogram for the spatial factor (urban green spaces).

The dendrogram for the spatial factor (Factor A) highlighted the existence of several similarity groups among the analyzed urban green spaces. Zones characterized by relatively close photosynthetic values tended to cluster together, indicating comparable ecological and structural conditions. In contrast, Zone 1 and Zone 10 displayed more distinct positions within the dendrogram, reflecting their markedly different photosynthetic performances compared to the other investigated green spaces (Figure 8).

**Figure 9:**
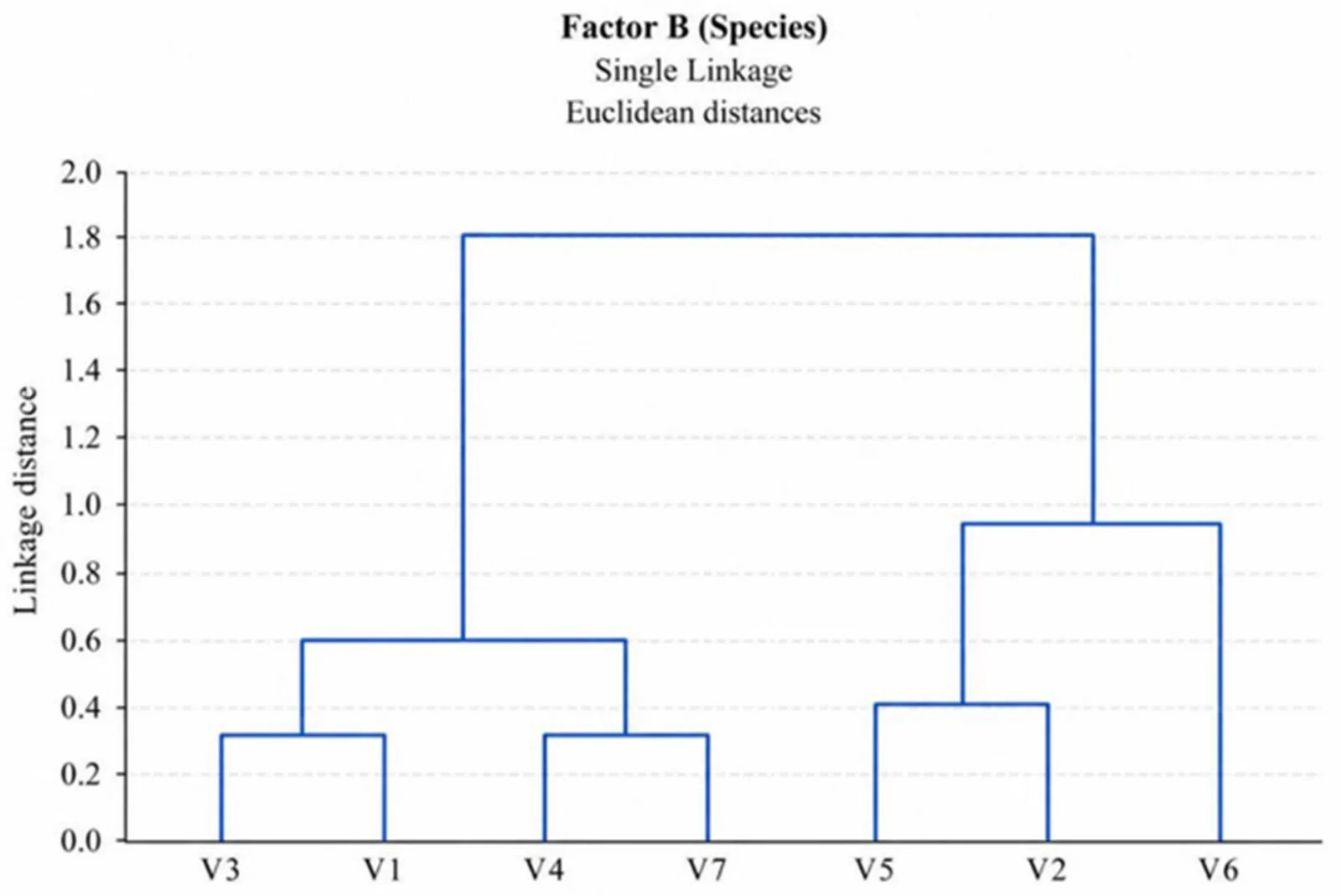
Cluster dendrogram for the species factor (plant variants).

The cluster analysis performed for the species factor (Factor B) also revealed differentiated grouping patterns among the analyzed plant variants. Variants presenting similar photosynthetic responses were grouped within the same clusters, suggesting physiological similarities and comparable ecological functionality. The clustering pattern was consistent with the statistical groupings identified by Duncan’s multiple range test (Figure 9).

Overall, the cluster analysis confirmed distinct ecological and physiological relationships between spatial organization and vegetation composition, supporting the hypothesis that green space composition significantly influences photosynthetic performance within the investigated campus ecosystem.

### 3.5. Comparative Interpretation of Photosynthetic Performance

**Figure 10:**
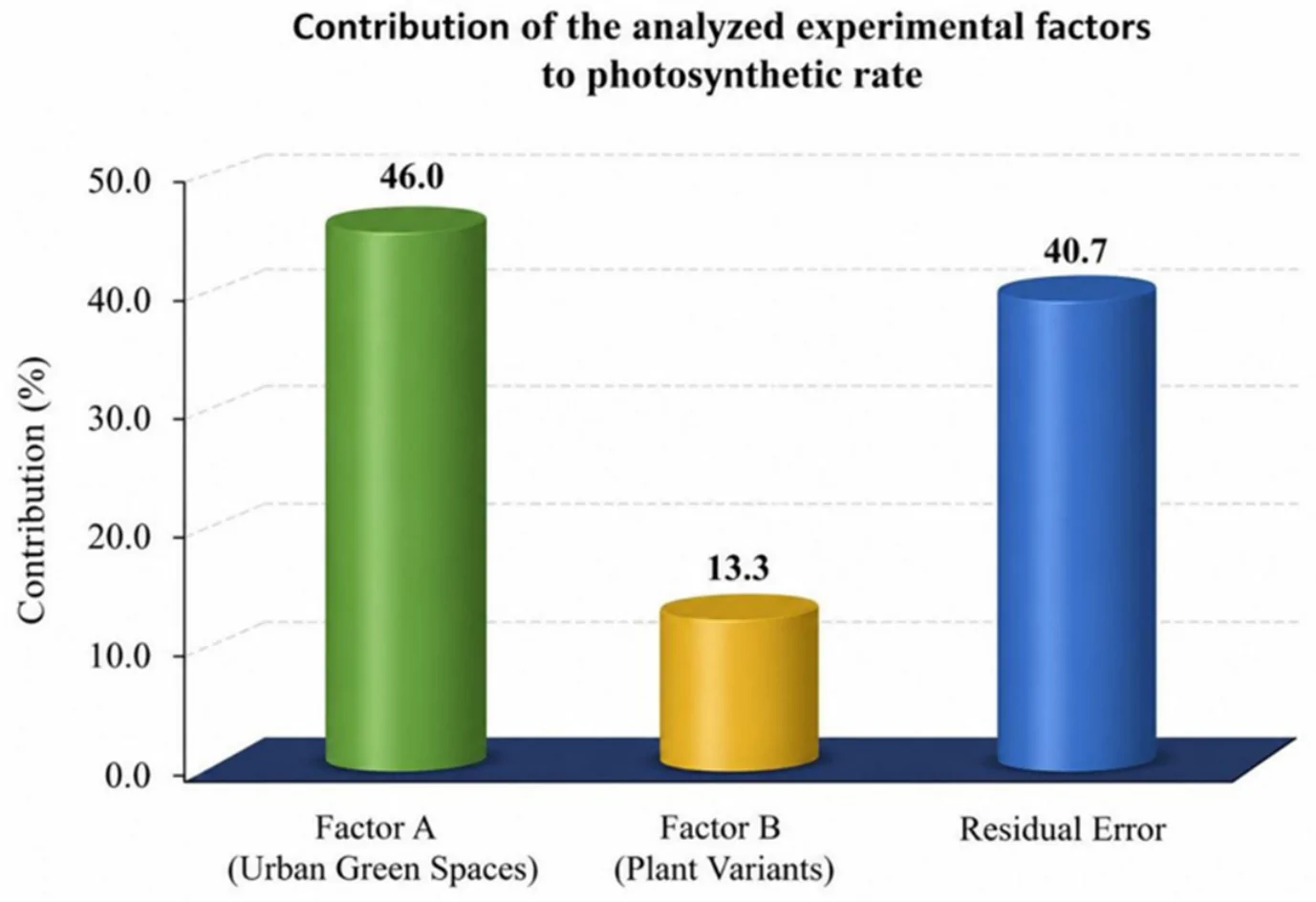
Contribution of the analyzed experimental factors to photosynthetic rate.

The comparative evaluation of the analyzed experimental factors demonstrated that the spatial factor (Factor A—urban green spaces) exerted the strongest influence on photosynthetic performance within the investigated campus ecosystem. According to the statistical analysis, Factor A contributed 46.0% to the variability in the photosynthetic rate, whereas the species factor (Factor B—plant variants) contributed 13.3% (Table 6 and Figure 10).

The analysis therefore indicates that the spatial organization and ecological characteristics of the analyzed urban green spaces exerted a substantially greater influence on photosynthetic functionality than the individual plant variants included in the experimental design. These findings emphasize the ecological importance of green space configuration, vegetation structure, and environmental heterogeneity in determining ecosystem-level physiological performance.

At the same time, the statistical error component accounted for 40.7% of the total variability, suggesting that additional environmental and ecological factors not included in the present study may also contribute considerably to photosynthetic behavior within urban ecosystems.

Overall, the two investigated experimental factors (spatial organization and plant variants) explained approximately 59.3% of the total photosynthetic variability recorded within the analyzed urban green spaces. These findings confirm the important contribution of vegetation composition and spatial configuration to the variability in photosynthetic performance within the investigated urban green spaces.

## 4. Discussion

Photosynthetic performance varied substantially among the analyzed urban green spaces within the campus ecosystem of the University of Life Sciences “King Michael I” from Timișoara. The results suggest that both the spatial configuration of urban vegetation and species-related variability contributed to photosynthetic performance, although their relative contributions differed considerably.

The statistical analysis revealed that Factor A explained 46.0% of the total variability in photosynthetic performance, while Factor B explained 13.3%. Together, the two analyzed factors accounted for 59.3% of the observed variability, whereas 40.7% remained attributable to residual variability and other uncontrolled environmental influences. These findings suggest that the ecological and structural characteristics of the urban green spaces themselves exerted a stronger influence on photosynthetic activity than species identity alone [3,7,10].

The marked contrast between Zones 1 and 10 was primarily associated with differences in vegetation composition and structural organization. Zone 1 was characterized by a relatively homogeneous assemblage composed exclusively of woody ornamental species, whereas Zone 10 integrated woody and herbaceous vegetation with different growth forms and ecological functions. In addition, Zone 10 represented the largest investigated green space and exhibited a more balanced woody–herbaceous distribution, suggesting greater structural heterogeneity and ecological complexity. These characteristics may have contributed to the higher estimated photosynthetic performance recorded in Zone 10. Environmental factors commonly associated with urban vegetation functioning, including solar exposure, irrigation regime, pruning intensity, and other maintenance practices, may also influence vegetation vitality and photosynthetic activity. However, because these variables were not directly measured in the present study, their contribution should be regarded as a plausible explanation rather than a demonstrated causal mechanism. Future studies integrating direct measurements of microclimatic conditions and maintenance intensity would provide a more comprehensive understanding of the factors governing photosynthetic performance in urban green spaces.

Among the analyzed zones, Zone 10 recorded the highest average photosynthetic rate (8.93), followed by Zone 9 with 8.29 and Zone 8 with 8.07. These values indicate that these urban green spaces were associated with higher estimated photosynthetic performance. Their more diverse vegetation structure and greater spatial heterogeneity may have contributed to improved ecological functionality and more favorable conditions for physiological activity.

Conversely, Zone 1 presented the lowest mean photosynthetic rate (5.93), indicating reduced physiological efficiency of the vegetation located in this area. Such reduced values may reflect lower structural complexity and less favorable site conditions, including increased anthropogenic pressure, lower vegetation density or microenvironmental limitations affecting plant metabolism.

The comparatively smaller contribution of species composition (13.3%) suggests that although physiological differences between species remain important, the urban environmental context may partially override species-specific responses. This observation aligns with studies emphasizing the dominant role of urban ecological conditions in determining vegetation performance and ecosystem functionality [3,5,7,8]. In highly modified urban ecosystems, plant physiological responses are often shaped more strongly by habitat quality and environmental stress than by taxonomy alone.

The residual variability identified in the analysis (40.7%) indicates that additional environmental and physiological variables not included in the present study likely contributed to photosynthetic performance. Future investigations should therefore adopt a more comprehensive methodological framework by integrating direct measurements of soil physicochemical properties, nutrient availability, soil moisture, microclimatic conditions, atmospheric pollution, and seasonal variability throughout the vegetation period. Standardizing field sampling protocols, expanding the range of investigated species and urban green spaces, and incorporating GIS-based spatial analyses together with remote sensing techniques would reduce unexplained variability and improve the robustness of the proposed evaluation model. Furthermore, validating the methodological framework across multiple urban environments characterized by different climatic conditions, vegetation structures, and management regimes would strengthen its transferability and support its application as a decision-support tool for sustainable urban green infrastructure planning.

Several research questions also remain open and deserve further investigation. First, it remains unclear to what extent the relative influence of spatial configuration and species identity varies under different climatic conditions or across seasons. Second, future studies should investigate the interactions between photosynthetic performance and additional environmental drivers, including soil physicochemical properties, nutrient availability, soil moisture dynamics, atmospheric pollution, canopy architecture, and microclimatic variability. The integration of repeated seasonal measurements, GIS-based spatial analyses, remote sensing techniques, and continuous environmental monitoring would contribute to the development of a more robust and transferable framework for evaluating the functional performance of urban green infrastructure. Such multidisciplinary approaches would also facilitate the validation of the proposed model across different urban ecosystems and support its application in evidence-based urban planning and landscape management.

Taken together, the results highlight the importance of strategic urban vegetation planning and ecologically informed management practices in maximizing the functional performance of campus green infrastructure. Urban green spaces designed with structurally diverse and ecologically functional vegetation may significantly enhance ecosystem services related to carbon sequestration, microclimate regulation and environmental resilience.

## 5. Conclusions

The study highlighted significant differences in photosynthetic performance across the analyzed urban green spaces within the campus ecosystem of the University of Life Sciences “King Michael I” from Timișoara.

The statistical analysis demonstrated that the spatial organization and ecological characteristics of the urban zones was the dominant factor influencing photosynthetic variability, explaining 46.0% of the total variation. Plant species composition contributed to a lesser extent (13.3%), while the remaining 40.7% was associated with residual environmental and physiological variability.

The highest photosynthetic performance was recorded in Zone 10, followed by Zones 9 and 8, suggesting that their structural characteristics were associated with higher estimated photosynthetic performance. In contrast, Zone 1 exhibited the lowest photosynthetic rate, indicating comparatively reduced ecological efficiency.

The findings further indicate that urban environmental conditions and landscape structure may exert a stronger influence on vegetation functionality than species composition alone. Consequently, the ecological design and management of urban green spaces represent essential components in improving the environmental performance of campus ecosystems.

Given the dominant contribution of spatial configuration to photosynthetic variability, the findings highlight the importance of incorporating vegetation structure into urban green space planning and management. Designing green spaces with diverse vegetation composition, balanced woody–herbaceous proportions, and greater structural complexity may enhance their ecological functionality and improve the physiological performance of urban vegetation. These findings support the integration of ecological criteria alongside species selection in the planning and management of sustainable urban green infrastructure.

The study contributes to the understanding of vegetation physiological responses in urban environments and supports the implementation of sustainable landscape planning strategies aimed at enhancing ecosystem services, environmental quality and urban resilience [10,12,15,22].

It should be noted that these findings are based on a single-campus ecosystem investigated under the temperate climatic conditions specific to Timișoara over one vegetation period. Consequently, the relative dominance of the spatial factor over species identity cannot be assumed to hold universally across urban ecosystems characterized by different climates, topographies and anthropogenic pressures. In ecosystems subject to more extreme climatic stress, steeper topographic gradients or markedly different pollution and land-use regimes, species-specific physiological tolerance may play a comparatively larger role, potentially narrowing or even reversing the balance observed in this study. The present results should therefore be regarded as evidence of the importance of spatial configuration within the specific ecological and climatic context investigated, rather than as a universally transferable hierarchy between spatial and species-related factors. Confirming the generality of this pattern will require comparative studies replicating the proposed methodological framework across urban ecosystems with contrasting climatic, topographic and anthropogenic profiles.

Future studies should integrate additional environmental variables, including soil properties, atmospheric conditions, seasonal dynamics and water availability, in order to provide a more comprehensive understanding of photosynthetic processes within urban green infrastructures.

## Figures and Tables

**Table 1:** Mean photosynthetic rate, standard error (SE), and 95% confidence intervals for the eight study zones.

Study Zone	Mean Photosynthetic Rate	SE	95% CI
Zone 1	5.93	0.369	5.03–6.83
Zone 2	7.50	–	–
Zone 4	7.86	0.357	6.98–8.73
Zone 5	7.21	0.214	6.69–7.74
Zone 7	7.43	0.071	7.25–7.60
Zone 8	8.07	0.429	7.02–9.12
Zone 9	8.29	0.510	7.04–9.53
Zone 10	8.93	0.561	7.56–10.30

**Table 2:** Student’s *t*-test analysis for the spatial factor (urban green spaces).

Zone	Photosynthetic Rate	Relative Value (%)	Difference from Experimental Mean	Significance
Zone 1	5.93	77.5	−1.72	***
Zone 2	7.50	98.0	−0.20	ns
Zone 4	7.86	102.7	0.20	ns
Zone 5	7.21	94.3	−0.40	ns
Zone 7	7.43	97.1	−0.20	ns
Zone 8	8.07	105.5	0.40	ns
Zone 9	8.29	108.3	0.60	ns
Zone 10	8.93	116.7	1.30	**
Experimental Mean	7.70	100.0	mt	—

DL 5% = 0.97; DL 1% = 1.30; DL 0.1% = 1.71; ns = not significant; ** = significant at 1% probability level; *** = significant at 0.1% probability level.

**Table 3:** Duncan multiple range test for the spatial factor (urban green spaces).

Zone	Photosynthetic Rate	Difference from Experimental Mean	Significance
Zone 1	5.93	−1.72	***
Zone 2	7.50	−0.20	ns
Zone 4	7.86	0.20	ns
Zone 5	7.21	−0.40	ns
Zone 7	7.43	−0.20	ns
Zone 8	8.07	0.40	ns
Zone 9	8.29	0.60	ns
Zone 10	8.93	1.30	**

Note: ns = not significant; ** = significant at 1% probability level; *** = significant at 0.1% probability level.

**Table 4:** Photosynthetic rate values recorded for the analyzed plant variants.

Variant	Photosynthetic Rate	Standard Error
V1	8.31	0.604577
V2	7.19	0.388880
V3	8.13	0.532430
V4	7.88	0.375000
V5	7.69	0.421916
V6	7.06	0.290282
V7	7.31	0.297872

**Table 5:** Duncan multiple range test for the species factor.

Variant	Photosynthetic Rate	Duncan Class
V1	8.31	A
V3	8.13	AB
V4	7.88	ABC
V5	7.69	ABC
V7	7.31	BC
V2	7.19	C
V6	7.06	C

**Table 6:** Relative contribution of the analyzed experimental factors to photosynthetic variability.

Experimental Factor	Contribution (%)
Factor A (Urban Green Spaces)	46.0
Factor B (Plant Variants)	13.3
Residual Error	40.7

## Data Availability

The original contributions presented in this study are included in the article. Further inquiries can be directed to the corresponding author.
